# Book Review: Faceworld: The Face in the Twenty-First Century

**DOI:** 10.1177/1329878X20949947a

**Published:** 2020-11

**Authors:** Karina Aveyard

This is a fascinating, highly engaging book that examines the history of the social and cultural construction of the face and its evolving role in identity, representation and communication. Primarily, Western in its orientation to history and philosophy, the book does much more than the title might initially suggest. Taking its starting point in the 19th century, it devotes significant attention to charting the shifting cultural and artistic practices and technological innovations and affordances that underpin contemporary emphasis on the face. This includes developments and fashions related to portrait painting, and later photography, the increased importance of the face as a primary form of identification and as an important aspect of 20th-century democratisation.

The second-half of the book is devoted more to contemporary development and deployments of the face and gives attention in particular to digital pathways of creation and dissemination in which the face occupies a central role, especially social media. The groundwork established by Zilio’s preceding historical survey provides a clear context for thinking about modern productions and representations of the face beyond popular perceptions of vacuous self-obsession. Instead, the book prompts us to reconsider many of the unconscious and naturalised ways in which societies have been encouraged to accept the modern centrality of the face as self-evident.

The book engages with some complex concepts and theories that draw on perspectives from multiple contemporary academic disciplines. This aspect of the text is excellent and it is an exemplar of the productive potential that lies in genuinely interdisciplinary approaches. The intellectual depth of this book is impressive, drawing on philosophical writing, creative practice and media and cultural theory from across several centuries.

At the same time, the text is accessible and straightforward and the logics of the arguments and philosophies is always clear, making this a book that could usefully be considered as a graduate level reading, as well as being important for scholars interested in social practice and the embodied nature of communication and representation. Given the complexities of the translation process, exactly how much and in what ways the clarity of the book is attributable to the skills of Robin Mackay is unclear (the book was originally published in French). However, his contribution should not be overlooked in considering the high quality of the reproduction of this text in English.

Karina Aveyard *University of East Anglia*

